# Modern Drug Laboratories

**Published:** 1887-11-19

**Authors:** 


### MODERN DRUG LABORATORIES.

BY OUR SPECIAL COMMISSIONER.

THE thought of pain and of the means taken to relieve it brings to the mind a strange series of pictures, as we review the first dawnings of therapeutic knowledge—the instinctive choice of some convenient plant, the half-superstitious sacrifice and consumption of some rare animal, and the shrewd if unphilosophical generalisations drawn from particular instances. Childishness and wisdom walked hand in hand: on reading one page of any ancient book on pharmacy, we laugh at the strange drugs mentioned in it, and wonder how sane human beings could ever believe in their utility; turning over the page, we wonder again how they came to know the use of materials which we of this conceited nineteenth century pride ourselves not a little on having rediscovered.

The South American savage has found out that a certain bitter bark will cure the fever and ague that haunt his luxuriant forests. The negro of Calabar knows of a bean that possesses wonderful—he thinks magical—qualities; it braces the soldier's nerves for battle, and if administered to anyone accused of a crime will, he says, kill the guilty and leave the innocent unscathed. Paracelsus, travelling to the East to find out the secret of the Philosopher's Stone, brings back with him a certain drug, which he calls "laudanum"; a sedative more powerful than any Europe has hitherto known. Some less famous alchemist, protected by his glass mask, prepares a powder for which a court lady will give him her richest jewels, because if she swallows a little of it, it will make her face fairer than that of any rival dame, and if it does not win for her the admiration she desires, she has only to administer a larger dose of that same powder to the lady who has aroused her jealousy, and it will send her to a land where earth's loves and hates and rivalries are unknown. The village witch goes out in the evening to gather the herbs whose secret virtues she knows—coltsfoot and foxglove, nettles and wood-sorrel, plucked at certain states of the moon, and prepared with much muttering of charms. Some day she will fail to cure, and then she will stand in some danger of fire and water—the stake or the horse-pond; but meanwhile she is, though hated, bribed and feared, and can laugh at the future danger.

Over all the ancient and mediæval medicine-making hung a veil of mystery. The powers of earth and air were called to help, not by the natural process of aiding growth in the animal or plant that was to be sacrificed to human necessity, but by some exercise of occult power, "mystic, wonderful," that impressed the imagination alike of pharmacist and patient. Methods of preparation were kept secret, and surrounded with unnecessary precautions. There were no patent laws in those days, and the only way to secure exclusive right in any specialty was to preserve an inviolable silence as to the mode of obtaining it. Valuable secrets thus died with the discoverers of them, and medical formulæ became a matter of inaccurate tradition.

Even after more systematic attempts were made to preserve accumulated knowledge, and to investigate the properties of those drugs whose value had hitherto been matter of hearsay, the method of making up prescriptions was wonderfully, even culpably, careless. Strange impurities crept into mixtures and ointments made by the doctor's boy or the untrained chemist's assistant; many of these did not materially affect the action of the constituent ingredients, but they certainly did not render the medicine more agreeable to the patient, and the doses of past days required no increase of nastiness. The tinctures prepared over the smoky surgery fire were guiltless of any added flavouring that would mitigate their bitterness; the powders might be tinged with soot and dust, but they remained as nauseous as ever. The pills and plaisters alone were not absolutely repulsive, even though some unknown anp undesired addition to the prescribed substances somewhat destroyed their usefulness. Even when the medicine was prepared accurately enough, some carelessness in the matter of affixing labels might result in the most untoward consequences.

It seems strange that a matter involving sometimes the issues of life and death should have been treated with such indifference to the comfort if not the well-being of the patient; that Dr. Bolus—with his scanty surgery furnishing, his cracked basin, thrown aside as unfit for kitchen use, by way of mortar, his dilapidated pestle, and spatula by no means scrupulously clean, his measuring glass stained with the mingled drugs of all his limited *répertoire* of prescriptions—should so long have been patiently borne as ruler of the destinies of sick humanities. There were rebels against his sway, sturdy topers who sang the praises of malt liquor and defied the doctor with their home-brewed ale. They had reason on their side in many cases; we, their descendants, recognise that now.

Dr. Bolus is well-nigh dead now. In remote districts he still can tyrannise in some degree; but most of us know that we can get our medicines in unobjectionable or even pleasant forms, and insist on having these. Within the last ten or fifteen years the improvement in the manufacture of drugs has been remarkable, and the manufacturers have succeeded in fulfilling the three requirements of the Latin saying, "*tute, cito, et jocunde*"—they are safe in administration, prompt in action, and, so far as may be, pleasant to the taste.

These reflections were aroused in our mind by a visit we paid to a drug factory at Wandsworth. The owners of it are Americans, and their preparations are marked by the ingenuity and neatness which are characteristic of our Trans-atlantic cousins. Not only are the drugs good in themselves, and prepared with scrupulous attention to accuracy of measurement, but they are attractive in appearance and inoffensive to taste and smell; some are even appetising.

Take, for example, Messrs. Burroughs and Wellcome's preparation of the Kepler Extract of Malt. In it we find all the tissue-forming and digestive qualities of beer or stout in a concentrated form, with the advantage that it is non-alcoholic, and so agreeable in taste that a youthful patient of our acquaintance was known to double the prescribed dose of his own accord. It is superior to malt liquors because of the diastase found in it, which is inevitably lost in the process of manufacture of beer, owing to the great heat employed; whereas the Kepler Extract is prepared at a temperature never exceeding 90 deg. F. The malt is moistened, warmed, dried, and concentrated, till the essence of the grain flows out as a rich brown syrup, which can be used alone, or is supplied united with cod-liver oil, hypophosphites, or other drugs, which the malt extract not only forms a convenient medium for administering, but also aids in assimilating. Considering how often cod-liver oil is refused by patients on account of its repulsive taste and indigestible qualities, the value of this solution of it in malt extract can hardly be exaggerated.

Still more interesting is the manufacture of those compressed tablets which in the case of some drugs are superseding lozenges, and in that of others are taking the place of pills. The average medicated lozenge is as unpleasant a thing as one need wish to meet. It is hard in the first place, and it requires some moral force as well as tooth power to crush it, with the certainty of meeting with an inharmonious combination of sweet and acrid tastes. The new tablets are prepared, according to the Wyeth process, of the desired drug alone, without any mixture to help adhesion, simply by the force of the pressure to which they are subjected. A measured quantity of the powdered drug which is to be formed into the tablet is allowed to fall into a round space of the desired size; an automatic movement of the machine puts it under a die, which comes down on it with a calm, silent, apparently gentle touch, that nevertheless changes the loose powder into a compact hard tabloid, covered with moisture. The powder seemed quite dry when it was put into the machine, but the sudden, severe compression brought to the surface an unexpected dampness. The tablets are then taken to the drying-room, where a considerable heat evaporates the moisture, before they are weighed, and packed in the bottles or cases in which they are sent out. There are over thirty varieties of these tabloids prepared, of various degrees of hardness. Those which are useful for their effect on the throat, like the well-known chlorate of potash tablets, are compressed very severely, and dissolve slowly; while those which act on the internal organs are made so as to dissolve speedily.

Besides these there are a number of hypodermic tabloids, intended for injection. As these are composed of drugs which are poisonous in large quantities, extreme care is taken in measuring the doses. The automatic machine is not used for the manufacture of these, where the hundredth part of a grain may make a serious difference in strength. The workman fills a tiny silver measure with the powdered drug, and empties it in the hollow beneath the stamp. By turning a handle he brings down the required pressure, and the tabloid is formed. It is obvious that, as a mere matter of convenience, these tabloids are preferable to the liquid form of the same drug. They are accurate in measurement; they dissolve easily in a little water; while, owing to their solid form, they are eminently portable, being not more than a fifth of an inch in diameter, and very thin. A tube containing a dozen of them would occupy hardly any space in the waistcoat pocket; and a case containing ten tubes of different tabloids, with silver hypodermic needle, and strong glass pestle and mortar for crushing the tabloids, is no larger than the massive card-case with which old-fashioned ladies arm themselves when they go out to pay afternoon visits. To a doctor whose practice extends over a large district such a contrivance is invaluable. To carry a bottle of morphia or tinct. digitalis over five or six miles of rough country road is decidedly a nuisance, which no man will submit to unless he is sure that he will be compelled to use it; but it is no trouble whatever to take a tube of morphine or digitaline tabloids, and very little to slip into one's pocket a flat case, of exceedingly moderate dimensions, containing all the requisites for the injection of a dozen medicines.

(*To be continued.*)

